# Industry 4.0 real-world testing of dynamic organizational life cycle assessment (O-LCA) of a ceramic tile manufacturer

**DOI:** 10.1007/s11356-022-20601-7

**Published:** 2022-05-13

**Authors:** Marco Cucchi, Lucrezia Volpi, Anna Maria Ferrari, Fernando E. García-Muiña, Davide Settembre-Blundo

**Affiliations:** 1 Gruppo Ceramiche Gresmalt, Via Regina Pacis, 136, 41049 Sassuolo, Italy; 2 Department of Sciences and Methods for Engineering, University of Modena and Reggio Emilia, 42122 Reggio Emilia, Italy; 3 Department of Business Administration (ADO), Applied Economics II and Fundaments of Economic Analysis, Rey-Juan-Carlos University, 28032 Madrid, Spain

**Keywords:** Industry 4.0, Organizational life cycle assessment, Manufacturing, Environmental impact, Sustainability

## Abstract

In manufacturing, Industry 4.0 operating models enable greener technologies. Thanks to digital technologies, environmental sustainability and organizational competitiveness are mutually reinforcing. The challenge for manufacturing organizations is to understand and quantify the magnitude of this synergistic action, and the holistic perspective of life cycle assessment tools may be a solution to the problem. Organizational Life Cycle Assessment (O-LCA) unlike Product Life Cycle Assessment (LCA) is still an under-researched methodology with few applications in manufacturing contexts. This paper aims to fill this gap by implementing and validating O-LCA in the case of an Italian ceramic tile manufacturer. Following the O-LCA guidelines and exploiting Industry 4.0 technologies to perform the inventory analysis, the environmental assessment was conducted in three different plants, comparing the sum of the partial impact results with the overall results scaled to the whole organization. The experimental results demonstrated the validity of the organizational approach as an appropriate methodological option to obtain relevant information on environmental performance that, being based on empirical evidence, better support decision-making processes. Furthermore, the study provides empirical evidence of how Industry 4.0 is an enabler not only for the adoption of greener technologies, but especially for facilitating the organizational environmental impact assessment that is the necessary condition in order to set up and maintain greener manufacturing contexts.

## Introduction

Manufacturing activities have long been associated with critical issues such as environmental pollution and climate change (Leong et al. 2019). More recently, a new approach has been emerging that could be defined as “*responsible industry*” (Hahn 2020), for which the manufacturing processes of products cannot disregard their environmental impact assessment. An important stimulus in this direction has come from the European Union’s ambitious plan launched in 2019 and called the *European Green Deal* (Bongardt and Torres 2022). It aims to achieve climate neutrality by 2050 through the transformation of the European economy, which will also have to be cost-efficient, fair and socially balanced. However, in order to ensure that the Green Deal objective does not remain an abstract concept with little quantitative definition, it is necessary for manufacturing companies to focus their attention not only on the individual environmental impacts of production (water footprint, energy consumption, atmospheric emissions), but to measure sustainability in a comprehensive way, analyzing the entire supply chain. In this sense, life cycle assessment (LCA) is a methodology that allows to identify, evaluate and quantify the environmental impacts of a product or process during its life cycle in an iterative way (Bisinella et al. 2021). In other words, from the sourcing of the raw materials for its manufacture to the end of its life, including the transport, manufacture and distribution of the product. The analysis of each of the phases makes it possible to identify both the resources used in the manufacturing processes (raw materials, semi-finished products, water, renewable and non-renewable energy sources) and the environmental impacts generated during production. This analysis allows to obtain an exhaustive knowledge of the environmental performance of the product or process analysed. The results of LCA thus help to identify opportunities for improvement, to provide relevant information in the strategic planning of products or processes, to establish priorities in design and/or redesign and to the selection of environmental performance indicators, which translate into competitive advantages at the industrial and corporate level (Pryshlakivsky and Searcy, 2021). Recently, the increasing digitalization of production processes and especially with the spread of the manufacturing model of Industry 4.0 has made available to companies large amounts of data, including environmental data, collected in real time (Favi et al. 2022). This has enabled the adoption of the LCA methodology in those companies that are more digitally advanced, precisely because of the simplification of data collection that can be made both automatic and dynamic (Ferrari et al. 2021).

The purpose of this study is to provide an empirical validation of the assumption that Industry 4.0 can be enablers of environmental impact assessment in a manufacturing environment by adopting an organizational perspective. The remainder of the paper is structured as follows. A brief theoretical background to introduce the relationships between sustainability, process digitization, and environmental impact assessment is provided in ‘Theoretical overview’ section. The ‘Materials and methods’ section details the application domain of the research and the methodological framework adopted. The ‘Results and discussion’ section describes to the readers the results obtained from the environmental assessment of the manufacturing organization, following the four steps provided by the LCA methodology. Finally, ‘Concluding remarks’ section concludes the paper by highlighting both the theoretical and managerial implications of the results obtained in the study, while also addressing its limitations and thus providing directions for future research.

## Theoretical overview

The topic of the relationship between Industry 4.0 (I4.0) and environmental sustainability plays a key role in both scientific and professional debates (Bai et al. 2020; Ghobakhloo 2020; Ejsmont et al. 2020; Sun et al. 2021). The concept of I4.0 was first used at the Hannover Fair in 2011 and, today, has entered business culture and legislation worldwide (Rauch 2020). The term I4.0 refers to the digitization process of companies (Dutta et al. 2021) which, supported by various government incentives (Cugno et al. 2021), aims to transform the organization of work to bring companies to manage physical and digital resources equally and in a single production system (Jiménez et al. 2021). Based on this approach, organizations now have a wealth of data and information at their disposal to help evaluate and optimize every aspect of their business, especially for sourcing phase (Fallahpour et al. 2021). It is therefore evident how I4.0 and digitalization allow to reduce waste and optimize the performance of processes with obvious and significant benefits for the environment and for the industry itself (Amjad et al. 2021). These wastes, if not minimized, go beyond the factory boundaries that generate them and affect the entire production chain, falling on the environment, economy and society. Embracing this change of perspective makes it possible to revolutionize the very concept of making industry, favouring and facilitating those processes that meet the principles of sustainability according to ESG (Environmental, Social and Governance) criteria (Huang et al. 2021). Therefore, for manufacturing companies, the transition to sustainable production systems is nowadays one of the main challenges to be consistent with the goals of the 2030 Agenda (Mathai et al. 2021). For this reason, companies aiming to comply with new environmental regulations, such as carbon emission policies (Chang et al. 2021), must design and implement natural resources (Barbosa et al. 2022) and energy (D’Adamo et al. 2020) sourcing strategies that ensure environmental protection while preserving their productivity, competitiveness, and profitability (Zhou et al. 2021). This requires tools that support evidence-based decision making, and the extensive collection of information and data needed to make better-informed decisions (Zhu 2020) and to design sustainable business models (Godina et al. 2020). Among the evidence-based tools that decision makers can use for environmental analysis (Douziech et al. 2021), the life cycle assessment (LCA) methodology is one of the most comprehensive for tracking and generating quantitative information about the environmental impacts of the activities and facilities involved in the production, supply, consumption and end-of-life treatment of products (Bisinella et al. 2021). Evidence of this is that the European Commission has promoted the adoption of the Product Environmental Footprint to harmonize the application of the LCA methodology by also integrating it with the Circular Footprint Formula (Schrijvers et al. 2021). However, the product perspective for assessing environmental impacts may not always be sufficient because product eco-design involves not only the productive functions of the company but also the managerial ones (Forin et al. 2019). Therefore, a comprehensive assessment of the environmental impact of the entire organization is necessary to effectively implement evidence-based decision making. Organizational life cycle assessment (O-LCA) addresses this need by extending the scope of LCA from the product life cycle to that of the organization and its value chain (Marx et al. 2020). However, the O-LCA as an increasingly relevant methodology within the broader theoretical approach of life cycle thinking (Toniolo et al. 2019) is still lacking examples of validation of the method in an operational context (Lo-Iacono-Ferreira et al. 2017; Manzardo et al. 2018; de Camargo et al. 2019), especially in manufacturing (Rimano et al. 2021). It is precisely in these factory contexts, where the relationships between digital technologies and sustainability are also under-researched (Beltrami et al. 2021), the applications of O-LCA in an Industry 4.0 environment are still unexplored. To fill the aforementioned gaps, this study aims to test and validate O-LCA by applying it to the sector that produces porcelain ceramic tiles (Almeida et al. 2016), an industry that is characterized by a long and complex value chain and intensive use of both energy and natural resources (Dondi et al. 2021). This research is carried out following the single case study approach as a methodological strategy (Horrillo et al. 2021), considering an Italian ceramic tile manufacturer that has already implemented Industry 4.0 digital technologies for manufacturing transition to circular economy (Vacchi et al. 2021), thanks to digitalization models of product LCA (Ferrari et al. 2021), product life cycle costing (LCC) (Medina-Salgado et al. 2021) and social organization LCA (SO-LCA) (García-Muiña et al. 2021).

## Materials and methods

### Context of application

The case study for the application of O-LCA regards a ceramic tile manufacturer that ranks among the top ten producers in the Italian ceramic industry. This is a very important sector for the European economy consisting of 133 manufacturing companies that in 2020 produced about 344 million square meters (m^2^) of tiles employing 18.747 people (Confindustria Ceramica 2020). The large production volumes make this a resource-intensive industry (Appolloni et al. 2022; Atılgan Türkmen et al. 2021) as shown by the specific consumption of production factors, which are on average: raw materials 20÷21 kg/m^2^, water 0.2÷0.3 m^3^/m^2^, electricity 6÷7 kWh/m^2^, methane gas 3÷4 Sm^3^/m^2^. The intensive use of resources (Slimanou et al. 2021) and the complex system of transport of large masses of raw materials (sourcing) and finished product (distribution) that characterize this industry, generate multiple environmental loads along the supply chain, making this context very promising for the validation of O-LCA in an operational setting.

**Fig. 1:**
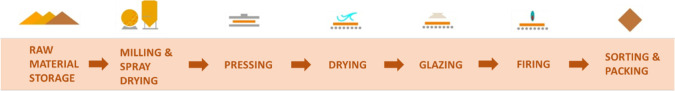
Ceramic tile production process (Elaborated from Mezquita et al. 2012)

The researched company produces porcelain stoneware tiles typology (Sánchez et al. 2019) of different sizes in its three factories. The production process starts with the procurement of raw materials. Raw materials (ball clays, feldspars, sands) are extracted from different parts of the world, either in non-EU territories (commonly Ukraine and Turkey) in Europe (e.g. Germany) or domestically and they are transported by land or ship directly to ceramic tile manufacturers. Once arrived, the materials are milled together with water in large mills to obtain a liquid substance called slip. The slip is sprayed with a flow of hot air to obtain an agglomerate of fine particles called spray-dried powder. From this moment onwards, the tile begins to take its characteristic shape. The spray-dried powder is conducted to the pressing stage, where it is pressed into the desired shape. The pressed and dried tile is then glazed and decorated using digital printers. Once decorated, the tile is fired at high temperatures (approximately 1200°) and, once fired, it can be subjected to further processing: cutting, rectifying, polishing, lapping. Rectification is used to produce perfectly squared tiles and cutting to obtain complementary formats (smaller) from the basic ones (larger). Polishing consists of the controlled removal of the surface layer by means of appropriate abrasive discs. Lapping is a finishing process consisting in performing an abrasion operation that provides the tiles with a fairly smooth surface but not completely shiny and reflective. Finally, tiles are then sent to the sorting line, which is mainly characterized by a size and flatness control unit, and they are finally packed. A simple scheme of the tile production cycle, inspired by the work of (Mezquita et al. 2012) is shown in Fig. 1.

After packaging, tiles are sold 75% through four commercial brands for the direct sales channel and the remaining 25% to the large-scale retail market. Once the tiles arrive at the customer’s destination, they are installed with the support of an expert and remain usable for a lifetime of up to 50 years. Upon disposal, part of the material is normally recovered for new purposes, e.g. road surfaces and concrete (Mangi et al. 2022).

For the purpose of this research, we will refer to the company’s factories as plant 1, plant 2 and plant 3. Plant 1 is the most productive plant in terms of finished products, producing around 10 million of the 20 million m^2^ produced by the company. Plant 1 is the most digitized plant in the group, where Industry 4.0 IoT technologies have already been implemented and extensively leveraged for the company’s environmental and socioeconomic impact analyses. From a production perspective, in plant 1, the spray-dried powder is produced for all three of the group’s plants. The raw materials arrive in the silos of plant 1 and from there the body milling and spray-drying process starts.

**Fig. 2:**
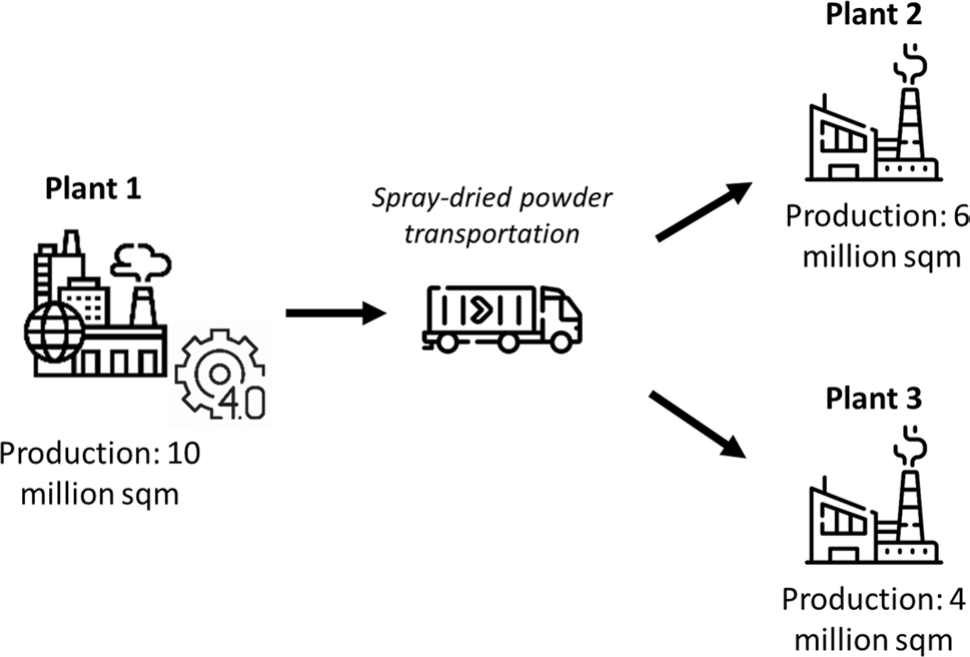
Company’s production plants layout. (Icons source: https://icons8.com/)

Plants 2 and 3 receive part of the spray-dried powder produced by plant 1 and they only undertake the remaining process steps from pressing to packaging, as illustrated in Fig. 2.

The three plants produce products in different formats and thicknesses. Plant 2 produces smaller format products (e.g. 20 cm × 20 cm). Plants 1 and 3 produce products of all sizes including the largest formats (e.g. 60 cm × 60 cm, 60 cm × 120 cm). Plant 3, in particular, produces thicker products resulting in a higher average kilograms per m^2^ of tile.

### Methodological approach

**Fig. 3:**
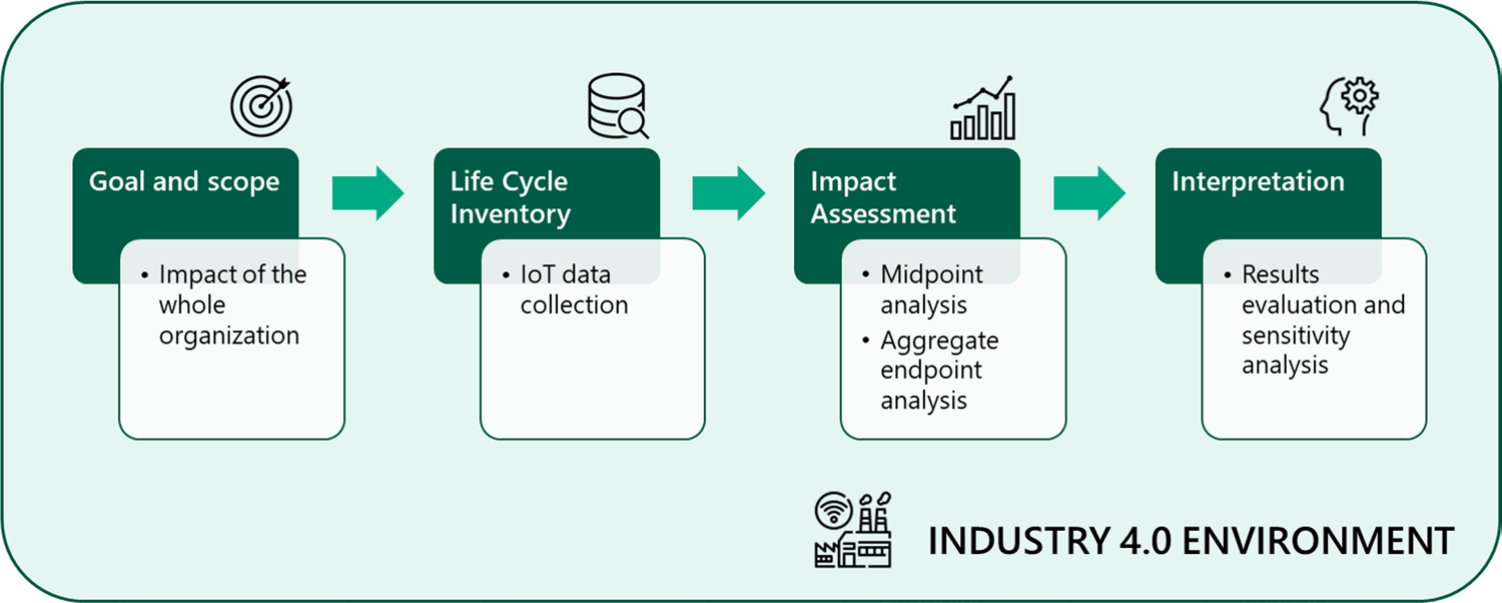
Schematic overview of the paper methodological approach^1^

The methodology applied in this study is based on the organizational life cycle assessment guidelines (Martínez Blanco et al. 2015) and covers the same steps as a LCA product analysis: goal and scope definition, life cycle inventory analysis, impact assessment, life cycle interpretation (Cremer et al. 2020) (Fig. 3).

For inventory analysis, the bottom-up approach was adopted. Following this approach, data should be collected to estimate the impact of each product in the organization (Martínez-Blanco and Finkbeiner 2018). Once the impact of each individual product is calculated, researchers should proceed by calculating the organization’s impact based on the production of a baseline time period. In this study, data collection was based on plant 1, one of the company’s three plants in order to obtain an environmental impact per functional unit of finished product. This impact, divided for each of the steps of the life cycle stages of the tile, from cradle to grave, is finally reproportioned to the output of the three plants to arrive at an organizational impact. This approach is therefore based on a simplified model, which derives the organization’s impacts from the inventory analysis of a single plant, thereby accelerating the time needed to collect inventory data and calculate impacts. In the last step of the analysis, due to the data obtained from the IoT technologies of industry 4.0 in the plant, the environmental impact of the organization is allocated on a monthly basis.

In order to offer a comprehensive analysis, in this study the impact assessment phase is conducted on two levels through a first and more detailed midpoint analysis and a second aggregate endpoint analysis (Rashedi and Khanam 2020). Both midpoint and endpoint analysis has been performed with the aim to provide more detailed results, thanks to the complementary of the two approaches. The former evaluates the magnitude of extractions or emissions at the beginning of the cause-effect chain throughout characterization factors that reflect more closely their relative importance; the latter considers the effects on specific damage categories (i.e. human health) as a result of aggregation and weighting of the results obtained from impact categories. This second approach is characterized by a higher level of uncertainty due to the need of making assumptions for the aggregation and weighting steps but on the other hand it provides environmental information that is easier to understand and manage also from decision makers and layman.

For the midpoint analysis, the following impact categories, taken from CML-IA baseline method, have been considered: global warming (kg CO_2_ eq.), acidification (kg SO_2_ eq.), eutrophication (kg PO_4_^3−^ eq.), photochemical oxidation (kg C_2_H_4_ eq.), abiotic depletion (kg Sb eq.) and abiotic depletion (fossil fuels) (MJ) (van den Heede et al. 2018). The impact categories selected are the same as those used in the environmental product declaration (EPD), a certification widely used by several ceramic companies and also provided at industry level by Confindustria Ceramica, the business association of Italian ceramic tile manufacturers (Confindustria Ceramica 2016).

A modified version of IMPACT 2002+ has been used for the endpoint results in terms of single score (points) (Ferrari et al. 2019). Furthermore, in order to evaluate the impact in terms of externalities that correspond to the environmental damage costs that the society is available to pay to restore the damage, EPS 2015dx method has been employed and the assessment results have been provided also in terms of Euros. The use of this double level of presentation of the results makes it possible to conduct a rather detailed analysis and monitoring of specific impact indicators covering the main environmental issues; at the same time, it provides two global results in terms of scores that are very useful for assessing the company's performance in an absolute sense and easier to understand and disseminate also to an audience not very familiar with environmental issues.

## Results and discussion

### Goal and scope definition

The analysis consists of calculating the impact of the whole organization from cradle to grave starting from plant 1, the plant with the highest level of industry 4.0 technologies and for which data collection is rapid and consolidated from previous LCA assessments. Assuming an identical process in all factories, we expect to derive the environmental impact per kilogram of tile from plant 1 and multiply it by the kilograms of tiles produced by the other two factories in order to obtain an impact for the whole production. This assumption is justified by the fact that all plants are equipped with the same technology and that, in recent years, the ceramic tile production process has become increasingly standardized (e.g. introduction of digital printing systems) (Jaramillo Nieves et al. 2020). The impact of all the company’s production from cradle to grave therefore allows the O-LCA analysis to be completed to a good degree of detail, excluding only the limited environmental impact of supporting activities (e.g. marketing, design, R&D activities). From a managerial perspective, the goal of O-LCA should be to support top management in the identification of environmentally critical phases and in strategic decisions concerning the introduction of new sustainability practices. The analysis should also aim to be used in external communication with stakeholders, for example through the elaboration of an organizational sustainability report.

**Fig. 4:**
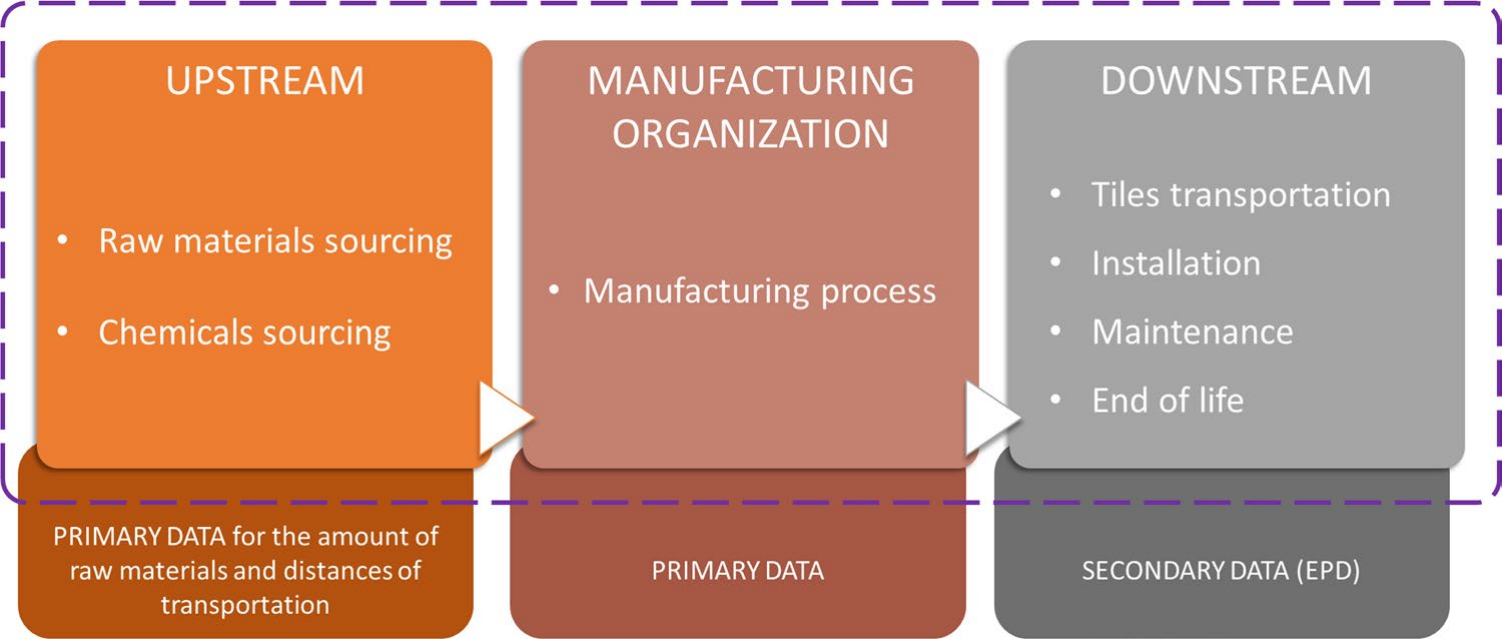
System boundaries and data quality proposed for the study

The scope of the analysis is to assess the environmental impact of the organization considering the entire life cycle of tiles produced, in a cradle-to-grave perspective; Fig. 4 shows the system boundaries with a schematic subdivision into modules and the related data used in the analysis. The reporting period for the analysis cover the year 2019. From 2020, the Covid-19 pandemic caused some production downtime, which is why it was decided to take 2019 as the most representative year to calculate the company’s impact. Although the inventory analysis is on the year 2019, due to IoT technologies, a monthly environmental impact will also be estimated on the year 2020. The functional unit we have decided to adopt is the kilogram of tiles, unlike the functional unit generally considered in this industry which is the square meter. Kilograms as a functional unit allows for consistent results across the three factories producing products of different sizes and thicknesses, overcoming the risk of underestimating the higher impacts of producing thicker tiles (Ferrari et al. 2021).

Regarding the system model, the attributional approach is followed to provide the environmental impact of the system without considering the effects of possible future changes in the demand of the analysed product (Weidema et al. 2018). Ecoinvent 3.6 database has been used for the modelling within Simapro 9.1.1.1 calculation code; several processes like recycling activities that are missing in the database have been created by the authors following the attributional approach with a 50:50 allocation in order to consider an equally shared responsibility between waste producer and user of secondary material (Appendix Tables 9,10,11,12,13,14,15).

### Life cycle inventory analysis

The Life cycle inventory analysis is carried out with reference to plant 1; the results of the environmental assessment from plant 1 are then used to estimate the impacts of plants 2 and 3.

The impact is calculated from cradle to grave, based on primary data for the gate-to-gate part and secondary data for the other phases. For cradle-to-gate data collection, the company uses a large amount of primary data (between 70 and 80%) made available by IoT industry 4.0 technologies (sensors and factory meters) linked to the company’s ERP (Ferrari et al. 2021). In order to complete the inventory analysis, we started from the analysis developed in previous research and supplemented it with some additional items. Below is Table 1 with all the primary data collected.

**Table 1:** Primary data inventory for O-LCA assessment

Inventory category	Inventory item	Measure unit
Raw materials sourcing	Extra-EU Clay	ton
	Extra-EU Na-Feldspar	ton
	EU Clay	ton
	National K-feldspar	ton
	National Sand	ton
	Recycled National Sand	ton
	Fluidizer	ton
Raw materials transportation	Extra-EU Clay	km
	Extra-EU Na-Feldspar	km
	EU Clay	km
	National K-feldspar	km
	National Sand	km
	Recycled National Sand	km
Chemicals sourcing	Glaze	ton
	Grit	ton
	Inks	ton
Chemicals transportation	Glaze	km
	Grit	km
	Inks	km
Other materials	Milling spheres and pebbles	ton
	Cardboard	ton
	Pallets	ton
	Other packaging materials	ton
Water consumption	Water from grid	m^3^
	Water from well	m^3^
	Recycled water	m^3^
Energy consumption	Electricity from grid	kWh
	Electricity from cogeneration	kWh
	Natural gas	m^3^
	Fuels	Litre
Waste production	Fired scrap	ton
	Wood/metal/plastic/paper waste	ton
	Hazardous waste	ton
	Other waste	ton
Atmospheric Emissions	Particulate Matter	ton
	Fluorine	ton
	Lead	ton
	Volatile organic compounds	ton
	Aldehydes	ton
	Sox	ton
	Nitrogen oxides	ton
	CO2	ton

The choice of these inventory data depends on a preliminary screening aimed at eliminating the inventory items with lower environmental impact, based on previous LCA elaborations. Previous assessments, which included a great number of product and process data, were necessary to evaluate the contribution of each variable to the environmental impact and then to make a choice based on an objective and robust criterion without the risk of losing reliability of the results. These preliminary analyses led us to exclude variables such as machineries, as they contribute less to the environmental loads than the rest of the variables. In any case, this exclusion was made to facilitate the analysis and then the implementation of the calculation algorithm in its first form; there is nothing to prevent these variables from being reconsidered and added to the analysis in the future once the company has fully mastered the tool.

Regarding the activities that occur out of the company gate, generic data are used and as recommended by the guidance on O-LCA, they follow recognized scenarios in particular those proposed by the sectorial EPD.

For the transport to the installation site, a distribution scenario in Italy, Europe and the rest of the world has been considered with the following assumptions:

- 300 km for national destination; truck with a capacity of 27 tons (51% of tiles sold);
- 1390 km for European destination; truck with a capacity of 27 tons (34% of tiles sold)
- 6520 km for transoceanic freight ship (15% of tiles sold).

Regarding the installation phase, a cementitious adhesive (6 kg/m^2^) has been included in addition to the end-of-life treatments of the tile packaging materials while for the maintenance during the entire life cycle (50 years) it was considered:

- 0.002 l/m^2^ of water;
- 0.0003 l/m^2^ of detergent;
- 2400 number of floor tile maintenance cycles within the life cycle.

Finally, the end of life has been modelled assuming that part of the tile is recycled, and part is landfilled.

### Life cycle impact assessment

The impact assessment is the calculation of the organization’s environmental impact based on the inventory data provided. According to the chosen methodology, an excel sheet with inventory data collection has been created. The assessment is divided into four phases:

1. *Unit impact of each inventory data item*: for each inventory data regarding plant 1 (see Table 1), the respective unit environmental impact is reported in terms of 8 categories; the first six are impact categories taken form CML-IA baseline method and provide the results according to the midpoint approach while the last two deliver aggregated single scores, respectively according to IMPACT 2002+ and EPS 2015dx methods. In a life cycle perspective, the impact of certain inventory items such as raw materials, is calculated by considering separately the extraction (or production) and transport of raw materials, in order to highlight separately these key phases.
2. *Total impact of each inventory data item*: once the unitary impacts of each inventory data are collected (phase 1), these impacts are multiplied by the quantities of each inventory data until a total value is obtained for each of the 8 categories considered. The result of these calculations provides, for the reference period selected, the total environmental impact for each of the individual inventory data considered in the study; the sum of these impacts consists in the global environmental assessment of the sourcing and manufacturing stages (from cradle to gate) of plant 1 for one year of production.
3. *Breakdown of impacts by process steps*: the environmental impact results of each inventory data is then divided by the following process phases: raw materials mining (body), raw materials transportation (body), chemicals production, chemicals transportation, milling and spray-drying, pressing and drying, glaze milling, glazing and decoration, firing, finishing and surface treatment, sorting and packaging, factory (other plant-wide impacts). The total impacts of each inventory data (phase 2) have then been allocated by process step (e.g. quantity of electrical energy used in milling and spray-drying, in pressing and drying, in glaze milling etc.). In order to provide a comprehensive overview of the impact within the entire life cycle of the tiles, also the phases beyond the gate of the company have been included in this phase, namely the distribution, installation, maintenance and end of life. Finally, all the impacts calculated per life cycle phase are then divided by the total kilograms of tiles produced by plant 1, in order to obtain an impact per kg of tiles.
4. *Extension of the results to other plants to obtain the organization’s impact*: by adopting a bottom-up approach, the impacts per kg calculated in phase 3 for company’s plant 1 can also be considered valid for the other two plants, assuming the same production process, same efficiency level and very similar equipment. By multiplying the impacts per kg by the total mass of tiles produced within the three factories, we obtained the environmental impact of the total quantity of tiles produced by the company.

Table 2 shows an extract of the phase 1 carried out with the example of energy consumption and the relative environmental results for the selected categories. In this specific context, the energy requirement for the manufacturing process is met by both the grid and a cogeneration plant that serves the entire factory and covers most of the demand. For the sake of clarity, we identify as *μ*_*i*, *α*_ the environmental unitary impact of each inventory item for each impact category, where *i* indicates the reference number of the inventory data and *α* indicates the impact category (e.g. GWP).

**Table 2:** Phase 1, environmental unitary impact per unit value of inventory item (μ_*i*, *α*_)—energy example

Inventory item (I.I.)	*i* (reference number)	GWP (kg CO_2_ eq)	ODP (kg CFC-11 eq)	AP (kg SO_2_ eq)	EP (kg (PO4) 3-eq)	POCP (kg C_2_H_4_ eq)	ADPE (kg Sb eq)	ADPF (MJ)	Points [Pt]	EPS (€)
Electricity from grid [1 kWh]	1	4.53E-01	6.73E-08	1.97E-03	5.60E-04	8.56E-05	1.46E-06	5.90E+00	1.74E-04	1.86E-01
Electricity from cogeneration [1 kWh]	2	1.77E-01	8.87E-08	8.41E-04	1.81E-04	6.91E-05	3.28E-08	9.64E+00	1.04E-04	2.92E-02
Natural gas [1 m^3^]	3	3.67E-01	2.33E-07	1.89E-03	1.71E-04	1.63E-04	5.00E-07	3.90E+01	4.06E-04	1.04E-01
Thermal energy from cogeneration [1 MJ]	4	3.10E-01	1.54E-07	1.47E-03	3.14E-04	1.21E-04	2.79E-08	1.68E+01	1.81E-04	5.24E-02
Fuels [1 ton]	5	3.66E+03	6.49E-04	2.85E+01	6.45E+00	5.67E-01	3.22E-03	5.04E+04	1.85E+00	2.14E+03

Once the impact unitary value of each individual inventory data is available (μ_*i*, *α*_), it is possible to proceed with phase 2 and calculate the total impact for each inventory item. We define with the variable *EI*_*i*, *α*_ the environmental impact, where *i* indicates again the reference number of the inventory data and *α* indicates the impact category. The calculation is represented by the following equation.

$${EI}_{i,\alpha }={Q}_i\times {\upmu}_{i,\alpha }$$

Where:

*EI*_*i*, *α*_ = total environmental impact (inventory item *i* and impact category *α*)

*Q*_*i*_ = total quantity of the inventory item *i* in the reference period

μ_*i*, *α*_ = environmental unitary impact (inventory item *i* and impact category *α*)

In this regard, considering the example of the electricity from grid (inventory item 1), the unit impact of the inventory item has been multiplied by the quantity of grid energy corresponding to one year of production (year 2019).

$${EI}_{1,\mathrm{GWP}}={Q}_1\times {\upmu}_{1,\mathrm{GWP}}$$

Following this calculation, we obtain the total impact of every single item of inventory.

Subsequently, in phase 3, the environmental impact results of the inventory data are divided by process phases. Firstly, for those data that are uniquely associated with a specific manufacturing step, the results have been linked to the respective stage such as for raw materials; on the other hand, those data that refer to more than one process (i.e. energy, emissions, etc.) have been split into percentages across the production steps, based on the information available to the company from IoT systems. In the example of electricity consumption, it is estimated that the most impactful phase is the body milling and spray-drying phase, with about 40% of the total factory consumption. At this stage, impacts from gate to grave based on secondary data are also added, in order to achieve a complete life cycle analysis. Once the impacts per phase are derived, the results is divided by the kg of tiles produced in plant 1 in order to calculate an impact per kg per phase for each of the 8 environmental impact categories considered, as reported in Table 3.

**Table 3:** Phase 3, impact of 1 kg of ceramic tile divided by process steps

Phase	GWP [Kg CO_2_ eq.]	ODP [kg CFC-11 eq]	AP [kg SO_2_ eq.]	EP [kg (PO_4_) ^3-^ eq.]	POCP [kg C_2_H_4_ eq.]	ADPE [kg Sb eq.]	ADPF [MJ]	Total Damage [kPt]	EPS [Pt = euro]
Body raw materials mining	1.88E-02	2.37E-09	8.48E-05	2.54E-05	3.40E-06	1.10E-06	2.46E-01	7.44E-09	5.43E-02
Chemicals production	2.66E-02	3.78E-09	1.28E-04	5.25E-05	1.01E-05	4.93E-07	3.25E-01	1.34E-08	5.09E-02
Body raw materials transportation	6.80E-02	1.10E-08	3.59E-04	9.21E-05	1.04E-05	1.14E-06	9.16E-01	2.90E-08	8.09E-02
Chemicals transportation	4.36E-04	7.98E-11	1.03E-06	2.27E-07	5.25E-08	1.09E-08	6.46E-03	1.68E-10	6.55E-04
Milling and spray drying	1.08E-01	1.24E-08	1.21E-04	2.36E-05	9.51E-06	5.50E-08	1.65E+00	2.93E-08	1.98E-02
Pressing and drying	5.53E-02	6.33E-09	6.22E-05	1.12E-05	4.87E-06	1.55E-08	8.45E-01	1.61E-08	1.02E-02
Glaze milling and glazing and decoration	1.46E-02	2.23E-09	2.55E-05	6.03E-06	1.85E-06	9.86E-09	2.39E-01	4.08E-09	2.84E-03
Firing	1.50E-01	1.43E-08	1.26E-04	1.68E-05	1.09E-05	3.08E-08	2.21E+00	3.64E-08	2.29E-02
Finishing and surface treatment	1.21E-02	1.85E-09	2.07E-05	4.76E-06	1.52E-06	4.41E-09	1.98E-01	3.16E-09	2.00E-03
Sorting and packaging	1.07E-02	1.12E-09	3.42E-05	1.19E-05	2.52E-06	2.05E-07	2.06E-01	6.36E-09	1.52E-02
Factory	1.20E-02	1.89E-09	2.98E-05	1.15E-05	2.45E-06	1.98E-07	1.69E-01	5.48E-09	1.78E-02
Transport to site	1.19E-01	2.15E-08	4.15E-04	7.40E-05	1.90E-05	2.84E-06	1.75E+00	4.67E-08	1.72E-01
Construction—installation	1.70E-01	6.03E-09	6.53E-04	1.73E-04	3.28E-05	1.07E-06	9.89E-01	5.04E-08	8.97E-02
Tiles maintenance	7.57E-02	3.81E-09	3.80E-04	2.92E-04	4.06E-05	2.25E-06	9.06E-01	1.05E-07	1.28E-01
End of life	1.83E-03	6.97E-10	2.23E-05	3.70E-06	7.68E-07	9.29E-08	4.65E-02	1.69E-09	6.42E-03

In phase 4, the impact per kg obtained in phase 3 is used with a bottom-up approach to calculate the impact of the whole production. By adopting this approach, the impacts per kg calculated for plant 1 (phase 3) can also be considered valid for the other two plants, based on the assumptions previously introduced. By multiplying the impacts per kg by the total mass of tiles produced within the three factories, it is possible to obtain the environmental impact of the organization. The impact of the organization for each process step is represented by the following equation:

$${OEI}_{p,\alpha }={EUI}_{p,\alpha}\times TP$$

Where:

*OEI*_*p*, *α*_ = organizational environmental impact (process phase *p* and impact category *α*)

*EUI*_*p*, *α*_ = environmental impact per kg of tiles (process phase *p* and impact category *α*)

*TP* = total production in kg of company’s three plants

Table 4 represents the results of the organizational assessment.

**Table 4:** Phase 4, O-LCA assessment (simplified model)—organizational impact assessment on three production plants

Phase	GWP [Kg CO_2_ eq.]	ODP [kg CFC-11 eq]	AP [kg SO_2_ eq.]	EP [kg (PO_4_) ^3-^ eq.]	POCP [kg C_2_H_4_ eq.]	ADPE [kg Sb eq.]	ADPF [MJ]	Total Damage [kPt]	EPS [Pt = euro]
Body raw materials mining	5.95E+06	7.51E-01	2.68E+04	8.03E+03	1.08E+03	3.49E+02	7.79E+07	2.35E+00	1.72E+07
Chemicals production	8.40E+06	1.20E+00	4.04E+04	1.66E+04	3.21E+03	1.56E+02	1.03E+08	4.23E+00	1.61E+07
Body raw materials transportation	2.15E+07	3.49E+00	1.14E+05	2.91E+04	3.30E+03	3.59E+02	2.90E+08	9.18E+00	2.56E+07
Chemicals transportation	1.38E+05	2.52E-02	3.26E+02	7.17E+01	1.66E+01	3.46E+00	2.04E+06	5.31E-02	2.07E+05
Milling and spray drying	3.40E+07	3.91E+00	3.83E+04	7.45E+03	3.01E+03	1.74E+01	5.22E+08	9.26E+00	6.28E+06
Pressing and drying	1.75E+07	2.00E+00	1.97E+04	3.54E+03	1.54E+03	4.91E+00	2.67E+08	5.10E+00	3.22E+06
Glaze milling and glazing and decoration	4.63E+06	7.05E-01	8.07E+03	1.91E+03	5.85E+02	3.12E+00	7.55E+07	1.29E+00	9.00E+05
Firing	4.74E+07	4.52E+00	3.97E+04	5.31E+03	3.44E+03	9.73E+00	6.98E+08	1.15E+01	7.23E+06
Finishing and surface treatment	3.83E+06	5.85E-01	6.53E+03	1.51E+03	4.81E+02	1.39E+00	6.26E+07	1.00E+00	6.31E+05
Sorting and packaging	3.40E+06	3.55E-01	1.08E+04	3.76E+03	7.97E+02	6.49E+01	6.51E+07	2.01E+00	4.81E+06
Factory	3.79E+06	5.98E-01	9.43E+03	3.65E+03	7.73E+02	6.26E+01	5.34E+07	1.73E+00	5.62E+06
Transport to site	3.76E+07	6.80E+00	1.31E+05	2.34E+04	6.01E+03	8.98E+02	5.53E+08	1.48E+01	5.44E+07
Construction—installation	5.39E+07	1.91E+00	2.07E+05	5.46E+04	1.04E+04	3.40E+02	3.13E+08	1.59E+01	2.84E+07
Tiles maintenance	2.40E+07	1.21E+00	1.20E+05	9.24E+04	1.28E+04	7.11E+02	2.87E+08	3.33E+01	4.06E+07
End of life	5.80E+05	2.20E-01	7.05E+03	1.17E+03	2.43E+02	2.94E+01	1.47E+07	5.36E-01	2.03E+06

The result obtained is representative of the whole company’s ceramic tile production and, in a broader perspective following the O-LCA guidelines, of the whole organization.

Starting from the annual results, it is possible, through the factory industry 4.0 IoT technologies, to reproportion the total organizational impact based on the kilograms of tiles produced by the company. This allows both to divide the impact into a smaller time unit (day, week or month) and to predict the organization's environmental impact based on the square meters of tiles the company intends to produce in the immediate future.

For the purposes of this research, we agreed to consider the impact category of ‘total damage (kPt)’, the most representative of the categories, and the time unit of one month. Based on these choices, we decided to assess the monthly impact of the 2019, year under analysis and to reproportion the impacts also over the 2020 year.

**Fig. 5:**
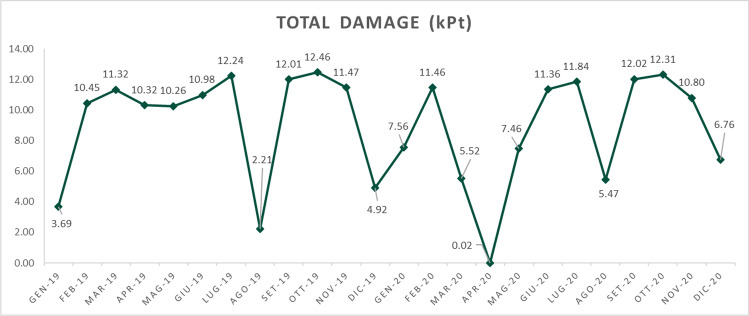
Monthly damage of the organization in kPt calculated using industry 4.0 IoT data

The results of the analysis are shown in the Fig. 5 below.

The graph clearly shows the evolution of the company's monthly environmental impact, which is subject to seasonality and decreases in proximity to the summer (August) and winter (December–January) plant closures. For the year 2020, we can also see the impact of Covid-19, which led all Italian manufacturing companies to a forced closure of plants in the March–April period and a slow recovery in the following months. In those months, in fact, the impact in kPt of the organization collapses to almost zero.

### Life cycle interpretation

In conclusion, the interpretation phase is intended to verify that the goal of the analysis has been achieved. The O-LCA analysis presented in this paper allows to calculate the environmental impact of the organization, considering the life cycle of the entire production of the company. Through Industry 4.0 IoT technologies, we then re-proportioned the environmental impact over the months of the year 2019 and extended the analysis to the year 2020 as well, showing the collapse of the environmental impact due to plant shutdowns.

**Fig. 6:**
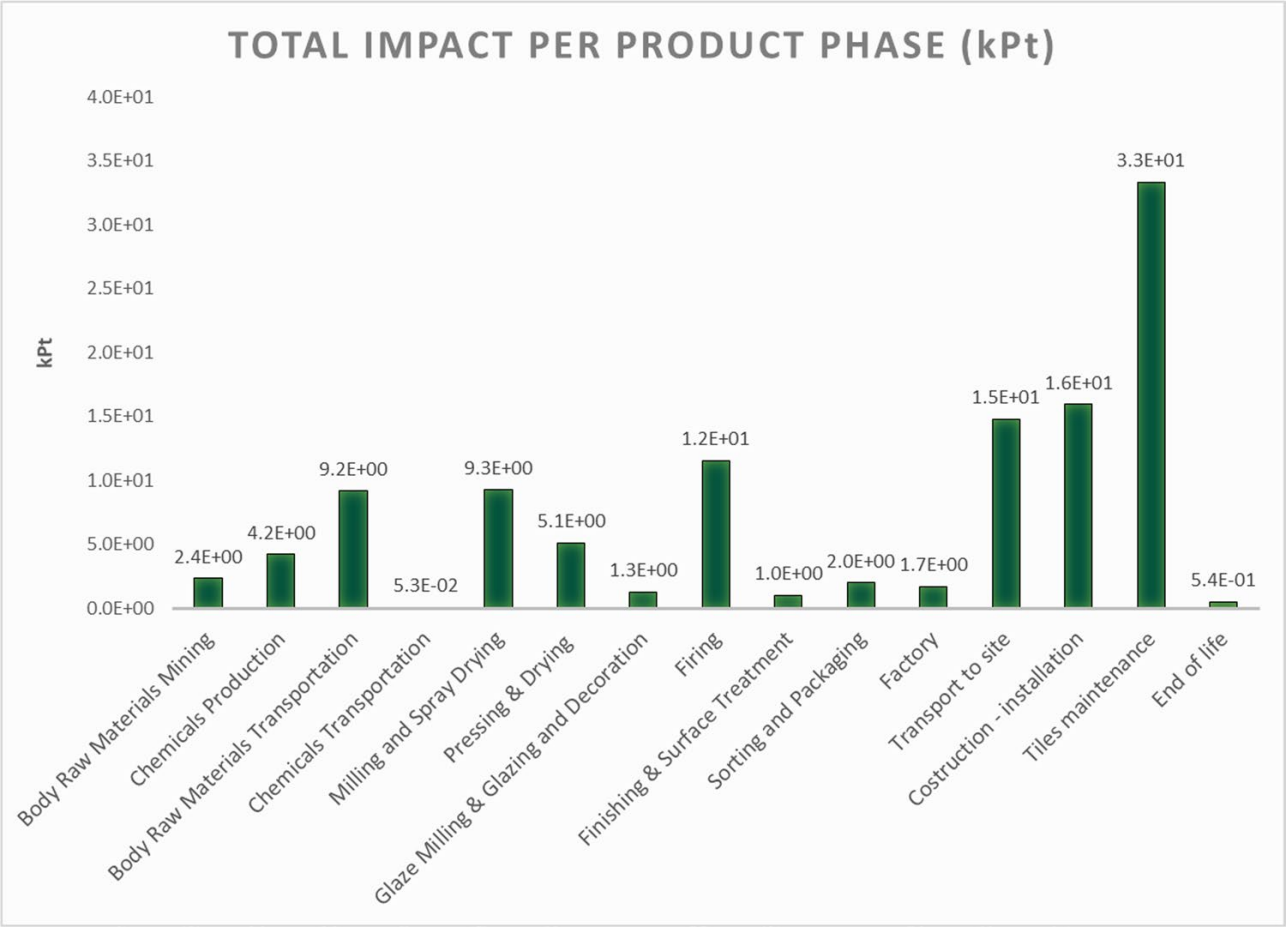
O-LCA assessment—impact of the organization with the simplified model (kPt)

As can be seen from Fig. 6, the results of the analysis, consistent with previous publications, show that the most impactful stages of the tile life cycle, in terms of points, are the maintenance, the installation by cementitious adhesive and the transport to the site (Pelton et al. 2016). The impact of the maintenance is obviously significant because it considers a weekly cleaning with water and detergent for 50 years, i.e. during the entire life cycle of the tiles so in a large time frame. The use of a cementitious adhesive is mainly responsible for the damage of the installation phase for which an amount of 6 kg/ m^2^ of adhesive is considered; regarding the transport to the building site, the impact is due to the transport of the tiles, again in accordance with the EPD, especially for European destination scenario that considers 34% of the tiles carried by trucks.

The impact throughout the life cycle and the information in Fig. 6 reveals some limitations of the analysis. The most evident limitation is that of the tiles maintenance phase, which impacts over a period of 50 years and results as the most impactful phase of the life cycle due to this assumption.

For the purpose of ensuring greater transparency, it was decided to reproportion the tiles maintenance phase by considering only one year, so as to make the impact proportionate to the other phases of the life cycle.

**Fig. 7:**
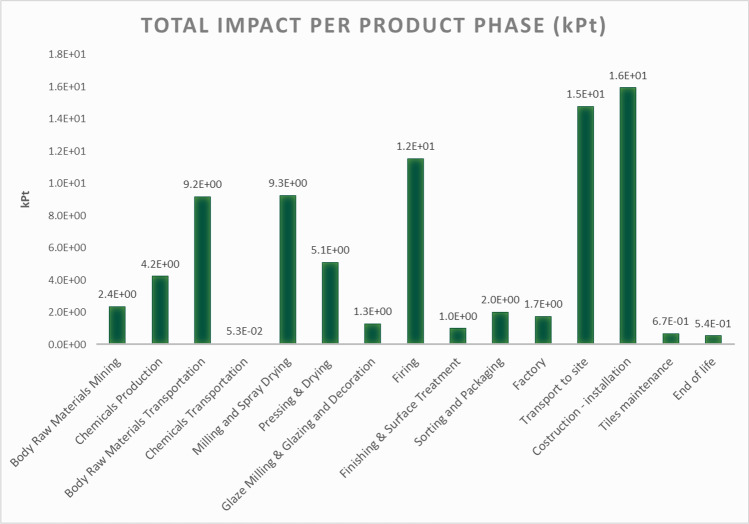
O-LCA assessment—impact of the organization with the simplified model considering one year of tiles maintenance phase (kPt)

The result is shown in the graph below in Fig. 7 and clearly indicates a downsized impact of the tiles manteinance phase, allowing to focus on other critical phases of the process such as raw materials and tiles transport, tiles firing and tiles installation.

**Fig. 8:**
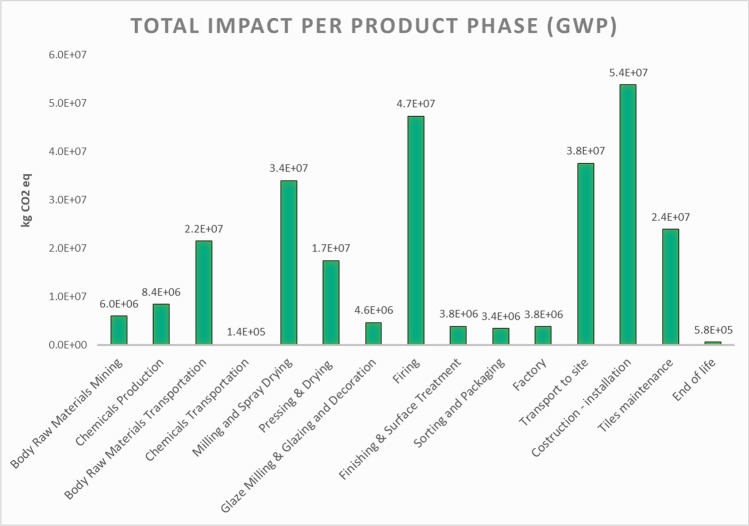
O-LCA assessment—impact of the organization with the simplified model (GWP)

Focussing on global warming impact category (GWP), the results reported in Fig. 8 highlight that the installation has the greatest impact, followed by firing, transport to the site, milling and spray drying, maintenance and body raw material transportation.

Within the manufacturing process, the most material- and energy-intensive phases are dominant, in particular the firing, the supply of raw materials and the milling and atomization. The considerable impact of the firing phase is mainly due to the consumption of natural gas burned in the kilns and the atmospheric emissions, especially CO_2_ emissions arising from the combustion; for the supply of the raw materials, the major contribution is attributable to the transports for a combination of long distances and impactful means of transportation (i.e. lorries) while regarding milling and atomization phase, as in the case of firing, methane consumption and CO_2_ emissions are primarily responsible for the damage, together with electricity consumption.

The bottom-up approach has been used in Table 4 to calculate the impacts of the company’s entire production starting from the impact of a single facility (plant 1), assuming in the first instance the same structure and level of efficiency for each factory (plants 2 and 3) and changing only the production and the weight per m^2^. Then to verify this assumption, a sensitivity analysis is conducted comparing the current simplified model with a detailed model based on a single inventory analysis for each of the three factories in which detailed data have been collected (Igos et al. 2019; Cherubini et al. 2018). The results of the detailed model are shown below in Table 5 and they are compared with those of the simplified model in Table 6.

**Table 5:** Phase 4, O-LCA assessment (detailed model)

Phase	GWP [Kg CO_2_ eq.]	ODP [kg CFC-11 eq]	AP [kg SO_2_ eq.]	EP [kg (PO_4_) ^3-^ eq.]	POCP [kg C_2_H_4_ eq.]	ADPE [kg Sb eq.]	ADPF [MJ]	Total Damage [kPt]	EPS [Pt = euro]
Body raw materials mining	1.66E+06	2.09E-01	7.47E+03	2.24E+03	3.00E+02	9.71E+01	2.17E+07	6.56E-01	4.78E+06
Chemicals production	2.32E+06	3.31E-01	1.12E+04	4.59E+03	8.87E+02	4.32E+01	2.84E+07	1.17E+00	4.45E+06
Body raw materials transportation	5.99E+06	9.72E-01	3.17E+04	8.11E+03	9.18E+02	1.00E+02	8.07E+07	2.56E+00	7.13E+06
Chemicals transportation	3.81E+04	6.98E-03	9.02E+01	1.98E+01	4.59E+00	9.58E-01	5.66E+05	1.47E-02	5.73E+04
Milling and spray drying	9.48E+06	1.09E+00	1.07E+04	2.08E+03	8.38E+02	4.85E+00	1.45E+08	2.58E+00	1.75E+06
Pressing and drying	4.05E+06	4.57E-01	7.05E+03	1.54E+03	4.11E+02	4.24E+00	6.39E+07	1.22E+00	9.26E+05
Glaze milling and glazing and decoration	6.64E+05	9.84E-02	2.88E+03	8.23E+02	1.26E+02	2.15E+00	8.64E+06	2.56E-01	2.74E+05
Firing	1.30E+07	1.34E+00	1.39E+04	2.77E+03	1.02E+03	5.62E+00	2.14E+08	3.56E+00	2.23E+06
Finishing and surface treatment	5.56E+05	8.25E-02	2.41E+03	6.88E+02	1.05E+02	1.80E+00	7.24E+06	2.13E-01	2.28E+05
Sorting and packaging	8.72E+05	7.77E-02	3.42E+03	1.16E+03	2.25E+02	1.92E+01	1.71E+07	5.41E-01	1.38E+06
Factory	7.69E+05	1.21E-01	2.96E+03	1.17E+03	1.99E+02	1.82E+01	9.17E+06	4.46E-01	1.59E+06
Transport to site	1.03E+07	1.87E+00	3.60E+04	6.42E+03	1.65E+03	2.47E+02	1.52E+08	4.06E+00	1.49E+07
Construction—installation	1.62E+07	5.66E-01	6.19E+04	1.62E+04	3.12E+03	9.99E+01	9.38E+07	4.77E+00	8.43E+06
Tiles maintenance	7.25E+06	3.65E-01	3.64E+04	2.79E+04	3.89E+03	2.15E+02	8.67E+07	1.01E+01	1.23E+07
End of life	1.62E+05	6.14E-02	1.96E+03	3.26E+02	6.77E+01	8.18E+00	4.10E+06	1.49E-01	5.66E+05

**Table 6:** Sensitivity analysis between detailed and simplified model

Phase	Δ detailed/simplified plant 2 [%]	Δ detailed/simplified plant 3 [%]
Body raw materials mining	0.00	0.00
Body raw materials transportation	0.00	0.00
Milling and spray drying	0.00	0.00
Chemicals production	− 0.66	− 17.96
Chemicals transportation	− 0.65	− 17.96
Pressing and drying	− 13.92	− 3.77
Glaze milling and glazing and decoration	− 28.70	− 13.28
Firing	10.93	10.95
Finishing and surface treatment	− 23.48	− 7.57
Sorting and packaging	− 3.42	− 6.47
Factory	− 7.59	− 2.42
Transport to site	− 1.42	− 0.06
Installation	7.51	− 12.46
Maintenance	8.61	− 12.34
End of life	0.00	0.00
Total	3.18	− 5.54
Total cradle-gate	− 0.60	− 0.25

The two models, simplified and detailed, are compared on the year 2019, considering the total damage express in points as representative of the total environmental impact of each process step. The table below shows the variation between the impact calculated with the detailed and simplified model, both for plants 2 and 3 and phase by phase.

For plant 2, the main variations occur in the phases of pressing and drying, glaze milling and decoration and finishing and they are all negative; this is mainly due to the fact that for plant 1, whose LCI data are the basis of the simplified model, the amount of particulates emissions and the electricity consumption per kilogram of tile are higher than those of plant 2 (which are considered in detailed model). Instead, regarding the firing phase, the impact variation is positive due to a higher methane consumption per kg in the detailed model with respect to the simplified one. In addition, it is useful to underline that the weight per m^2^ of the tiles in plant 1 is higher than that of the tiles produced in plant 2, so it means that tiles in plant 1 are thicker. This difference emerges in the results of the installation and maintenance; in fact, for these phases, the starting impact is calculated with reference to 1 m^2^ of tile, so for 1 m^2^ the impact is the same in both plants. Therefore, in order to obtain the damage of 1 kg of tile, this impact is divided by a higher value of kg/m^2^ for plant 1 compared to plant 2, so the final result is lower for plant 1 and the differences in the table are positive. For plant 3, the trend of the variations is very similar to the previous one. The impact change related to the chemicals used for the glazing and decoration is greater due to a lower consumption per kg of these materials in the detailed model compared to the simplified one. It should be also noticed that the sign of the variation for the installation and maintenance phase is reversed with respect to the previous situation because in this case the weight per m^2^ in the detailed model is higher than that considered in the simplified model (in plant 3 thicker tiles are manufactured). Finally, it is important to emphasise that in plant 2 not all finishing treatments are present while in plant 3 there is none; consequently, the differences in this phase arise from the fact that the simplified model replicates plant 1 as it is with, assuming all the same manufacturing steps for the other two. In addition, it should be noted that the highest differences in absolute terms occur in stages that contribute little to environmental damage (i.e. finishing and surface treatment, glaze milling and glazing and decoration, chemicals production and transportation, etc.) so considering the overall impact their relevance is minor.

Regarding the change in the damage of the entire life cycle of the tiles, the differences in the percentages are low (3.18% for plant 2 and − 5.54% for plant 3); these values are further reduced if the cradle to gate impact is considered (− 0.60% for plant 2 and − 0.25% for plant 3). Therefore, it can be concluded that the simplified model that assumes three equal plants just with different production and weight per m^2^, provides LCA results phase by phase that are similar from those obtained with the detailed model. Instead, considering the total impact, both with a cradle-gate and cradle-grave approach, the differences are greatly reduced; the simplified model can thereby be used for accounting for the global organization impact with a good degree of accuracy.

## Concluding remarks

The results of this study offer both theoretical and practitioner implications.

From a theoretical point of view, the validation of O-LCA through experimentation in an Industry 4.0 context filled part of the gaps highlighted in the literature. Moreover, the results have shown that in environmental assessments, it is essential to broaden the perspective of analysis considering the entire life cycle of the product, including not only the manufacturing phases, but also the sourcing of resources, the distribution of the product and its use and end of life. The organizational approach of the O-LCA, which embraces the entire value chain, helps to improve the quality of data for inventory analysis because it can stimulate collaboration between economic agents in the supply chain by encouraging the sharing of increasingly accurate primary data, including through industrial symbiosis relationships. As an effect of this virtuous behaviour, the environmental responsibility of each actor in the value chain becomes evident and transparent, motivating organizations to pursue continuous improvement in their environmental performance.

From the practitioners’ perspective, the validation of O-LCA in a manufacturing context has shown how this environmental assessment model can be seen as an effective tool to support evidence-based decision making. The method’s organizational approach helps the path of companies towards quantitative determination of their environmental performance, while also providing relevant information for non-financial reporting and transparency to stakeholders. In fact, the product perspective, characteristic of LCA, can make data collection for inventory analysis more difficult, especially for those companies that have large and deep product assortments and are produced in separate manufacturing plants. Therefore, the O-LCA can be the first step in defining the organization’s environmental baseline, preparatory to single product category-specific LCAs. The results of this study also show the sustainability-enabling potential of Industry 4.0 digital technologies. Industry 4.0 technologies demonstrate a twofold benefit: on the one hand, they allow for rapid inventory data collection and thus enable the model to be replicated on subsequent years with little effort. On the other hand, they allow for analysis to be provided on time units that are much narrower than the year and also allow for the calculation of predictive impacts based on kilos of tiles produced.

However, even organizations with lower levels of digital maturity can successfully implement and benefit from the use of O-LCA.

Despite the originality of the application of the O-LCA, the model shows some limitations. First of all, as explained in the O-LCA guidelines, for a complete evaluation of an organization the model should also consider supporting activities, i.e. activities not directly involved in the production process (e.g. marketing, design, R&D activities). Another limitation concerns the application of O-LCA in a single product context (glazed porcelain stoneware tiles) in a company with three similar production plants. A different and more fragmented context would make the analysis significantly more complex, forcing scholars to further simplifications that could lead to unreliable results. Finally, a last limit concerns the difficulty of replicability and transferability of the model in other companies, limit already highlighted in the O-LCA guidelines. Despite this limitation, the model is still extremely relevant in the internal assessment of the environmental impact of the organization over time.

## Figures and Tables

**Fig. 9:**
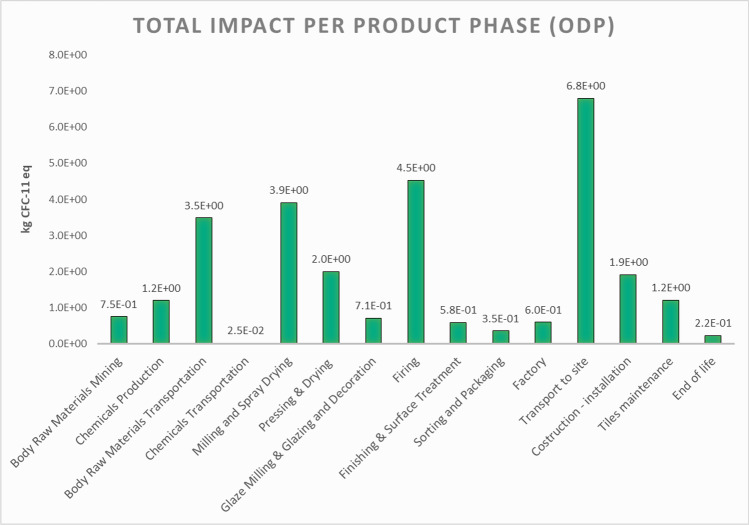
O-LCA assessment—impact of the organization with the simplified model (ODP)

**Fig. 10:**
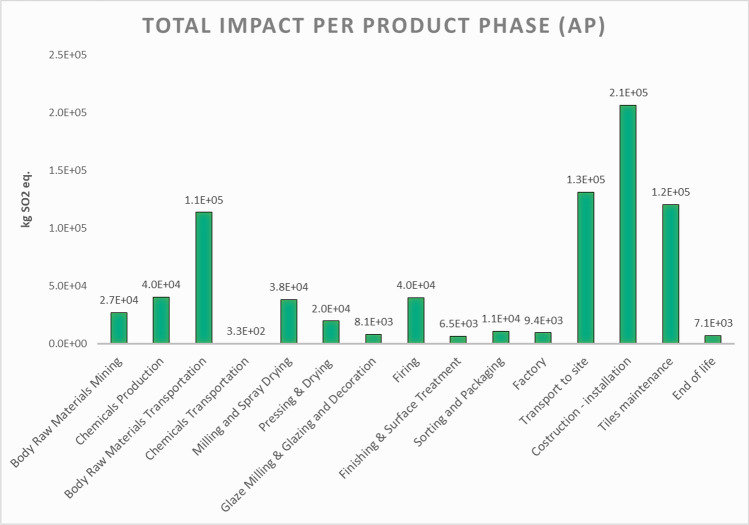
O-LCA assessment—impact of the organization with the simplified model (AP)

**Fig. 11:**
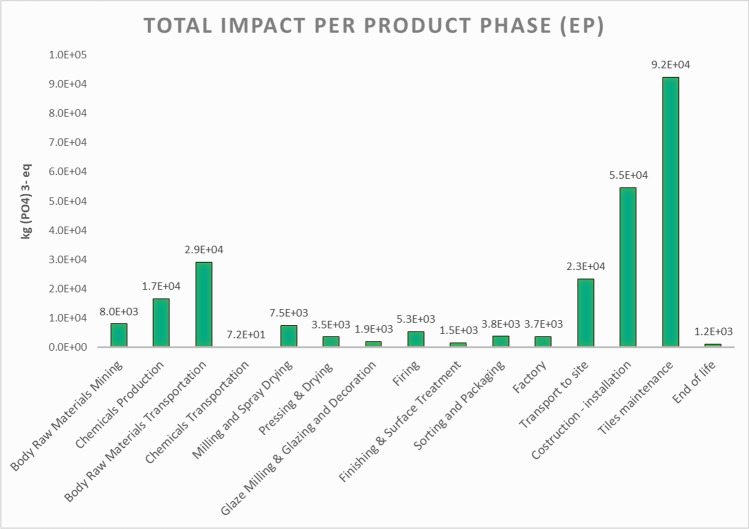
O-LCA assessment—impact of the organization with the simplified model (EP)

**Fig. 12:**
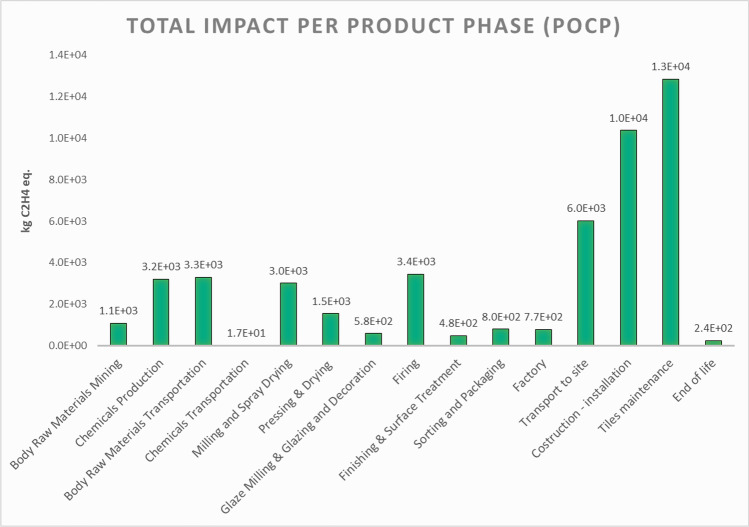
O-LCA assessment—impact of the organization with the simplified model (POCP)

**Fig. 13:**
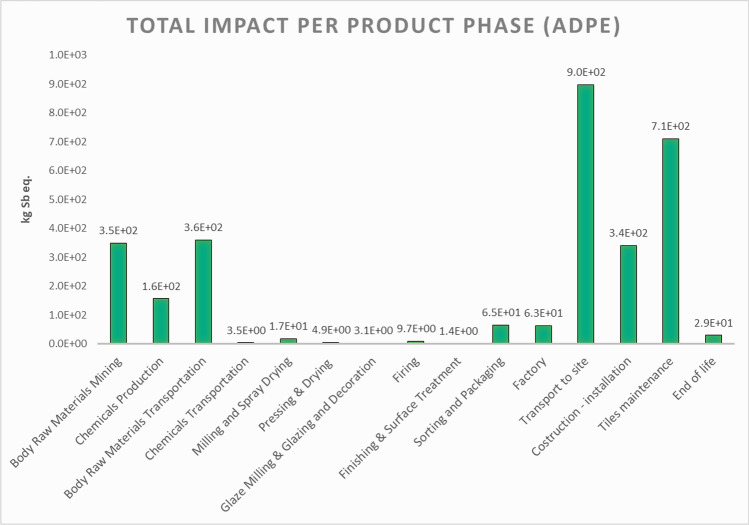
O-LCA assessment—impact of the organization with the simplified model (ADPE)

**Fig. 14:**
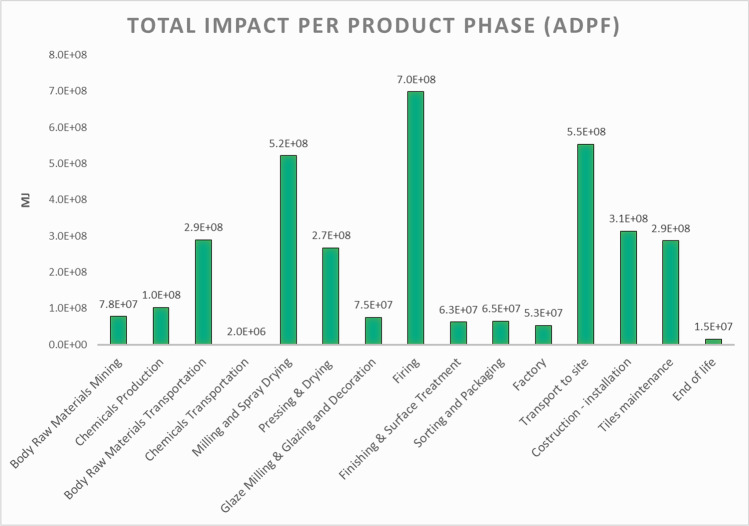
O-LCA assessment—impact of the organization with the simplified model (ADPF)

**Fig. 15:**
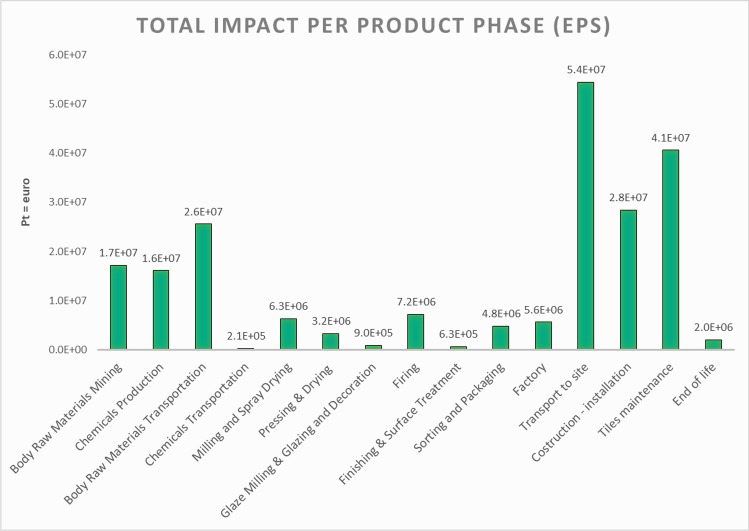
O-LCA assessment—impact of the organization with the simplified model (EPS)

## Data Availability

The primary datasets used in this study are available from the corresponding author by reasonable request. While all data processed or generated in the impact assessment are included in this article.
